# Prediction of Right Heart Failure in LVAD Candidates: Current Approaches and Future Directions

**DOI:** 10.3390/jcdd12070240

**Published:** 2025-06-23

**Authors:** Frederick Vogel, Zachary W. Sollie, Arman Kilic, Ethan Kung

**Affiliations:** 1 Department of Bioengineering, College of Engineering, Computing and Applied Sciences, Clemson University, Clemson, SC 29634, USA; 2 Department of Surgery, Division of Cardiothoracic Surgery, Medical University of South Caroline, Charleston, SC 29425, USAkilica@musc.edu (A.K.); 3 Department of Mechanical Engineering, College of Engineering, Computing and Applied Sciences, Clemson University, Clemson, SC 29634, USA

**Keywords:** right heart failure, Michigan score, EUROMACS score, MCRN score, ALMA score, CRITT score, right ventricular longitudinal strain, pulmonary artery pulsatile index, physiology simulation coupled experiment, mechanical circulatory support, VAD

## Abstract

Right heart failure is a condition where the right ventricle fails to pump blood into the pulmonary artery, and, in turn, the lungs. This condition frequently presents after the implantation of a left ventricular assist device (LVAD). Ventricular assist candidates who have LVADs implanted possess various pathophysiological and cardiovascular features that contribute to the later development of RHF. With LVADs serving as bridge-to-transplantation, bridge-to-candidacy, and destination therapies, it is imperative that the pre-operative indicators of RHF are identified and assessed. Multiple predictive models and parameters have been developed to quantify the risk of post-LVAD right heart failure. Clinical, laboratory, hemodynamic, and echocardiographic parameters have all been used to develop these predictive approaches. RHF remains a major cause of morbidity and mortality after LVAD implantation. Predicting RHF helps clinicians assess treatment options, including biventricular support or avoiding high-risk surgery. In our review, we noted the varying definitions for RHF in recent models, which affected respective predictive accuracies. The pulmonary arterial pulsatile index (PAPi) and right ventricular longitudinal strain parameters were noted for their potential to enhance current models incrementally. Meanwhile, mechanistic and machine learning approaches present a more fundamental shift in the approach to making progress in this field.

## 1. Introduction

Many cardiovascular disorders culminate in heart failure with varying etiologies that guide their coordinated treatment. In 2023, approximately 6.7 million Americans had heart failure with more than eight million predicted to have developed the condition by 2030 [1]. Unfortunately, these high rates of pathology do not scale with the availability of viable donor hearts. According to the US Department of Health and Human Services’ Organ Procurement and Transplantation Network, over 4500 hearts were transplanted nationally in 2024. While this number was a 24% increase from 2020, many patients remain on the heart transplant waitlist [2]. For patients in advanced heart failure, heart transplantation remains the best treatment.

However, with the prevalence of heart failure and the scarcity of available hearts for transplantation, LVADs have been used as an alternative strategy, with over 2500 implanted in 2022 [3]. These important mechanical support devices allow patients to either live long enough to receive a heart transplant or, for those who cannot receive a heart, serve as a destination therapy. 

LVAD implantation can result in multiple complications which can affect the health and life of a potential patient, such as infection, gastrointestinal bleeding, pump thrombosis, and stroke. Notably, right heart failure (RHF) remains one of the most impactful complications of patient mortality. This paper will seek to review past foundational studies, analyze the current predictive models for RHF, and assess proposed novel parameters and techniques to advance predictive accuracy. These predictive models use varying combinations of preoperative echocardiographic, hemodynamic, laboratory, and clinical parameters to indicate the future development of RHF post-LVAD implantation. Given recent advances in speckle-tracking echocardiography, composite hemodynamic parameters, and machine learning, a review is needed to consider these developments in the field. Since RHF prediction is most clinically valuable preoperatively—when interventions such as medical optimization, temporary mechanical support, or surgical modifications can be implemented early for optimal outcomes—this review focuses solely on preoperative parameters. This paper will seek to understand how the literature has evolved with the innovation of LVAD devices and the direction to which the field should progress

## 2. LVADs and Post-LVAD Right Heart Failure

### 2.1. Left Ventricular Assist Devices (LVADs)

Since the first implantation of LVADs in the 1960s, LVADs’ use, development, and survival rates have significantly increased. LVADs create additional parallel blood flow, directed through implanted cannulas that support the flow from the native ventricle [4]. During the first iteration of LVADs, this supplemental blood flow was pulsatile, mimicking the behavior of the left ventricles (LV). These devices, however, were large, noisy, and susceptible to infection (e.g., HeartMate XVE, HeartMate VE, Thoratec PVAD). The second iteration of LVADs, developed during the 1990s, were continuous-flow pump devices which were small, quieter, and less susceptible to infection (e.g., HeartMate II). This improvement was further advanced by the third and most recent iteration of LVADs, which boast significantly smaller sizes and longer device life spans. These devices are characterized by their centrifugal flow, facilitated by their magnetically driven and suspended impeller that minimizes shear (e.g., HeartMate 3) [5]. Previously, fully implantable LVAD systems were unrealized due to inefficiencies in conventional wireless charging and the size of second-generation LVADs. Yet, with recent advances in power electronics and centrifugal-flow LVADs, these systems are now being considered with some in active development [6]. However, these systems are in early development, with none in the clinical trials stage.

This transition from pulsatile-flow (PF) to continuous-flow (CF) LVADs has improved surgical outcomes and complications, but some researchers have noted changes to the hemodynamics of LVAD recipients. Kato et al. observed more effective unloading of the LV in PF compared to CF LVADs through BNP and ECM biomarkers’ metrics and echocardiographic measurements of LV function [7]. Previous animal studies showed that continuous unloading of the LV with CF LVADs altered the physiologic profile of myocardial and vascular hemodynamic energy utilization, compared to PF LVADs, which better preserved normal physiologic values [8]. Other researchers like Garcia et al. have shown no statistical significance of LV unloading and hemodynamic improvement; however, their study did not study CF or PF LVADs as groups but solely compared the HeartMate XVE to the HeartMate II [9]. As differences between parameters’ predictive efficacies for RHF have been observed between PF and CF patient populations, this review has elected to separate the predictive models based on their respective LVAD cohort type, enumerated either as majority pulsatile-flow or majority continuous-flow.

Due to the durations over which patient data was collected, all studies reviewed, including the earliest Utah, Michigan, and Pennsylvania scores, had a cohort of patients with CF LVADs [10,11,12]. Therefore, studies with over fifty percent PF LVADs were classified as the majority pulsatile-flow group; whereas, studies with over fifty percent CF LVADs were classified as the majority continuous-flow group. Due to the field’s precipitous transition from PF to CF LVADs, only the CRITT score reported similar quantities of PF and CF patients (58.7%, 41.3%) [13]. All other studies were greater than seventy percent for one type of LVAD over the other. The CRITT and Kormos et al. scores were grouped independently due to their unique patient population and the absence of echocardiographic data [13,14], respectively. Modern parameter studies were exclusively or over eighty percent of CF patients and thus classified as the majority of CF.

### 2.2. Definitions of Right Heart Failure

RHF is characterized by the inability of the right ventricle to support optimal circulation during the presence of adequate preload [15]. While multiple disorders can result in the development of RHF, post-LVAD RHF will be the focus of this paper. Since the partnership between INTERMACS and the National Heart, Lung, and Blood Institute, hospitals, and industry, more clinically relevant definitions have been formed [16]. In 2008, INTERMACS defined RHF using two hemodynamic factors: central venous pressure > 18 mmHg with a cardiac index < 2.0 L/min/m^2^. Since then, several definitions have been developed, including Matthew et al.’s, defining right ventricular failure as the need for postoperative intravenous inotrope support for >14 days, inhaled nitric oxide for ≥ 48 h, right-sided circulatory support, or hospital discharge on an inotrope [11]. The majority of predictive models have based their definitions around similar criteria to Matthew et al.’s definition of RHF; however, there is some deviation. As Scalligitol et al. recognize, the inclusion or exclusion of inotropic support or inhalation of NO into a study’s definition is the main discongruence [17]. Stratified definitions for RHF (acute, early, and late) were also rarely applied in the literature reviewed. Increased clarity surrounding the infusion duration and use case for inotropic agents should be incorporated into RHF definitions to reduce selection bias, as these qualities vary across clinicians and healthcare centers. An overview of all reviewed studies’ definitions can be found in Table S1.

### 2.3. Limitations of Comparing Models

Direct, head-to-head comparison of models based solely on area-under-the-curve (AUC) values is inherently problematic due to the presented models’ heterogeneity in LVAD types, RHF definitions, patient populations/datasets, and study eras. While we report AUC values as presented in the original studies, meaningful comparisons require consideration of each model’s development, validation, parameters, and context. This paper aims to provide relevant details for each model to support a more informed interpretation of AUC comparisons.

### 2.4. Clinical Impact of Accurate RHF Prediction in LVAD Patient Care

The accurate prediction of post-LVAD RHF carries profound clinical implications, directly influencing patient management strategies across the perioperative continuum. Identifying patients at high risk for RHF preoperatively allows clinicians to implement targeted optimization strategies, which may include aggressive medical management of volume status and pulmonary vascular resistance or, in select cases, temporary mechanical circulatory support to “bridge” the right ventricle [18]. Intraoperatively, this foreknowledge can guide anesthetic choices, fluid management, and preparedness for immediate RV support. Postoperatively, for those identified as high-risk, clinicians may proactively institute measures such as pharmacological RV support (e.g., inotropes like milrinone, dobutamine) or inhaled pulmonary vasodilators (e.g., nitric oxide, epoprostenol), and maintain a lower threshold for considering temporary RV mechanical support (e.g., percutaneous RVADs, ECMO) [18,19,20]. Critically, for individuals with a prohibitively high predicted risk of RHF, these predictive insights can fundamentally alter the therapeutic pathway, prompting consideration for upfront biventricular support (BiVAD) or a total artificial heart, or in some circumstances, a decision to defer LVAD implantation in favor of continued medical management or alternative advanced therapies if deemed too high risk for isolated LVAD support.

## 3. The Right Ventricle Anatomy and Pathophysiology

### 3.1. Anatomy of the Right Ventricle

The right ventricle of the heart pumps blood from systemic venous return through the pulmonary valve and into the pulmonary artery. Due to the interplay between the right and left ventricles, the anatomy of both ventricles is important for understanding post-LVAD RHF. Significant differences between the two ventricles include operating pressures, volumes, and myocardial thickness.

While the right ventricle contains a larger blood volume, the left ventricle’s myocardial mass is approximately six times that of the right ventricle [21]. Additionally, the right ventricular wall is about one-third the thickness of the left ventricular wall [21]. These differences result from the organization and structure of the muscle fibers that drive each ventricle’s pumping function. The right ventricle operates at systolic and diastolic pressures of approximately 25 mmHg and 8 mmHg, respectively, whereas the left ventricle functions at significantly higher pressures.

Ventricle–ventricle interactions are primarily dictated by two factors: circulatory interdependencies and pericardial influences. Circulatory interdependencies have been studied through testing how the loading conditions of one ventricle impact the systolic and diastolic function of the other [22]. Ex vivo animal studies have shown that LV contractility is augmented by increases in right ventricular pressure and contrariwise with left ventricular pressure modulating right ventricular contractility [23,24]. Deviations in volume and pressure cause these ventricles to remodel by adapting to these changes. Due to the discrete pericardial space, hypertrophy or distension of one ventricle can compress the adjacent ventricle [25]. Pericardial constraint has also been shown to induce hemodynamic deterioration of the right ventricle during both pressure and volume loading [26]. The consequences of these ventricle–ventricle interactions are intrinsically tied to the pathophysiology exhibited in post-LVAD RHF patients.

### 3.2. Pathophysiology of Post-LVAD Right Heart Failure

While the specific mechanism that links LVAD implantation to RHF is still unknown, several physiological mechanisms have been proposed to explain this increased incidence of RHF. Left ventricular remodeling, pericardial disturbances, and interventricular septal shifting are the primary mechanisms that are being investigated in the current literature. These pathophysiological mechanisms stem from the increased left ventricular outflow inherent to LVAD implantation. This increased output flows through the circulatory system and induces a higher preload in the right ventricle. While the right ventricle can adjust to preload variation through the Frank–Starling mechanism, the volume overload due to increased left ventricle output causes deleterious results, such as right ventricular distension and tricuspid annular dilatation [27,28]. The distension of the right ventricle can cause increased pericardial strain and induce a shifting of the interventricular septum, while tricuspid changes (increases in annular dilation, papillary muscle displacement) and right atrial enlargement can increase tricuspid regurgitation and right ventricular preload [29,30].

The pericardium contains the heart and preserves the morphology of the ventricles, especially under high preload [31]. Pericardial strain induced by high preload of the right ventricle can be exacerbated by pericardial damage created during LVAD surgery. The surgical sternotomy common to contemporary LVAD surgery has been connected to poor pericardial health and decreased right ventricular ejection fraction [32]. Left anterolateral thoracotomy has been shown to decrease the effect cardiac surgery can have on right ventricular function; however, only a minority of LVAD patients are operated on using this approach.

The interventricular septum is a muscular wall that separates the ventricles. Post-LVAD implantation, pathologic shifting of the interventricular septum can occur, especially in LVADs with poorly prescribed pump speeds [33]. Mouratoglou et al. linked the duration of septal displacement toward the left ventricle during a complete cardiac cycle to an increased right ventricle volume, mass, and decreased ejection fraction [34]. This decreased ejection fraction is compounded by intrinsic RV dysfunction existing prior to implant. The inability to compensate for the geometric and volume load changes predisposes the right heart to worsening decompensation.

Further, afterload of the right ventricle plays a key role in right heart strain. Many patients exhibit elevated pulmonary pressures as a downstream effect of an increased postcapillary wedge pressure due to the failing left ventricle. Preoperatively, right heart catheterization paired with the application of pulmonary vasodilators tests the reversibility of pulmonary vascular resistance in the event of unloading of the left ventricle [28]. In the event of native pulmonary vascular disease, a persistently elevated PVR provides a strain that can be difficult for the right ventricle to overcome when combined with the insults of geometrical changes that occur with the LVAD device.

Finally, there is an important differentiation between early and late-onset RHF following LVAD implantation. Late-onset RHF has several definitions, but, in summary, it occurs in the following: the need for initiation of right-side mechanical support >30 days after LVAD implantation, readmissions with RHF symptoms and the need for additional diuresis or inotrope support >30 days after implantation, delayed increase in end-organ dysfunction, or reduction in pump flows by >30% without tamponade [35]. The definition of early vs late right heart failure has been an involving subject. Consequently, the reported prevalence of late RHF following LVAD implantation varies from as little as 1% to as high as 44% [28,35]. To standardize these definitions, organizations like the International Society for Heart and Lung Transplantation (ISHLT) have updated their definition of early post-implant RHF to be more comprehensive. In their statement paper, they defined early post-implant RHF as the need for implantation of a temporary or durable RVAD (including ECMO) within 30 days following LVAD implantation for any duration of time, a failure to wean from inotropic or vasopressor support or inhaled nitric oxide within 14 days following LVAD implantation, or having to initiate this support within 30 days of implant for a duration of at least 14 days [36]. ISHLT suggests primary diagnosis of RHF through the presence of at least two clinical findings (Table 1). Additionally, ISHLT highlights RHF’s association with the tabulated manifestations in aiding diagnosis (Table 2). With the introduction of a more comprehensive definition, the field may come to an agreement over defining early and late RHF [36].

### 3.3. Risk Factors That Correlate with RHF

Ever since RHF was linked to LVAD implantation, researchers and clinicians have sought to improve LVAD treatment assessment by predicting RHF in prospective patients. Multiple testing modalities have been applied to LVAD patients, providing quantitative measurements that correlate with RHF: laboratory, clinical, hemodynamic, electrocardiographic, echocardiographic, cardiac MRI, and cardiac CT parameters. Due to the retroactive design of many of the first prediction studies, parameters were limited to those commonly obtained during conventional LVAD prescreening. Early studies with PF patient populations conducted univariate and multivariate analyses on these available parameters, and hemodynamic, clinical, and laboratory parameters were found to have the greatest link at this time. Hemodynamic parameters such as right arterial pressure, right ventricular stroke work index, and pulmonary arterial pressure were of particular interest during this time [37,38,39]. While these studies were used to inform future predictive models later discussed, their impacts were limited by their disparate, small patient populations and dissimilarity to modern CF patient populations. Of the parameters that were of prominence in these early papers, the right ventricular stroke work index has had the most sustained interest [12,37,39,40]. The compound hemodynamic measurement quantifies the workload and contractility of the right ventricle based on the mean pulmonary arterial pressure, mean central venous pressure, stroke volume, and body surface area of the patient. Regardless, the relevance of this parameter and others previously identified have been reduced in more modern models with more comprehensive CF patient populations [41,42] In Bellavia et al.’s meta-analysis, the right ventricular stroke work index was found to be a strong discriminator in PF patient populations; however, this impact was substantially smaller in CF patient populations [43]. These evolutions reflect the field’s ability to conduct studies on larger CF populations with more available echocardiographic and hemodynamic data. It is important to recognize that the structural and functional parameters used in these assessments are manifestations of the underlying disease processes, representing the downstream pathophysiological consequences of diverse cardiac etiologies. These quantifiable parameters capture the common pathways through which different underlying conditions—whether ischemic, dilated, or other cardiomyopathies—ultimately affect cardiac structure and function, making them valuable for risk stratification regardless of the initial cardiac insult. Since patient populations have shifted to almost exclusively CF LVADs, parameters that were prognostically useful in PF patient populations must be reevaluated and replaced with parameters and predictive models more applicable to CF patients.

## 4. Predictive Models of Post-LVAD Right Heart Failure

### 4.1. Majority PF Patient Population Models

Following the interest in predicting post-LVAD RHF, researchers and clinicians have sought to create standardized predictive models to assess preoperative risk. Matthew et al.’s study is one of the oldest and most referenced predictive score models for right ventricular failure [11]. This study relied on three laboratory values and one clinical value for predicting risk: aspartate aminotransferase, creatinine, bilirubin, and preoperative vasopressor use. While these values expressed a respectable AUC of 0.73, the study’s patient cohort greatly differs from current ventricular candidates. This study used University of Michigan patients who received LVADs between October 1996 and August 2006. Eighty-six percent of these devices were first-generation PF devices. As discussed earlier, implantation of these devices is almost never performed now due to their inferior size, noise, and infection risk compared to second- and third-generation CF LVADs [4]. 

Regardless, many researchers continue to use Matthew et al.’s risk score as the benchmark metric, commonly referenced as the Michigan score in the literature [10,11,13,14,40,41,44,45,46]. 

Of the earlier PF models compared to the Michigan score, Wang et al.’s decision tree risk method is the only model to report a significant increase in AUC of 0.87 [44]. This approach was driven by the C4.5 decision tree algorithm, one of the first examples of machine learning applied to post-LVAD RHF prediction. Since the patient population of this study was majority PF (77.6%), future papers like Loghmanpour et al. referenced this paper but did not apply this algorithm to their dataset for comparison [46].

### 4.2. Bridge Models–The CRITT and Kormos et al. Scores

The CRITT Score was the second benchmark study after the Michigan score to be widely used for comparison. This study had a unique LVAD patient population with similar amounts of PF and CF patients (CF = 41.3%, PF = 58.7%). This predictive score model was also the first to incorporate echocardiographic parameters as predictive factors [13]. Clinical, hemodynamic, and echocardiographic parameters were used to create the CRITT score: [preoperative intubation, inotrope use, right ventricular dysfunction diagnosis], [central venous pressure], and [tricuspid valve regurgitation severity, tachycardia], accordingly. This model boasted an AUC of 0.80, which compared to the Michigan score that had an AUC of 0.61 on this dataset [13]. Atlrui et al., the researchers who created the CRITT score, aimed to create a score that could quickly and accurately assess the risk of RHF. The unique Boolean weights of the model allowed for bedside-ready calculation. This quick calculation would be useful to the respective half of LVAD recipients who would be in cardiogenic shock [47]. This study bridged the clinical shift from PF to CF LVADs, and introduced echocardiographic parameters into multivariate studies for RHF risk. Currently, the Michigan score and CRITT score serve as the field’s most ubiquitous benchmark for prediction.

The Kormos et al. study was one of the earliest predictive models to be trained with an exclusive CF patient population [14]. Like the later majority CF models [41,42], parameters such as CVP/PCWP and blood urea nitrogen were used to predict post-LVAD RHF. However, despite being an exclusively CF study, echocardiographic parameters could not be used due to the lack of echocardiographic patient data. This contrasts against all later majority CF studies, which had access to and incorporated echocardiographic data into their models [40,41,42]. On a patient population size of 484, they reported an AUC of 0.68, which was similar to previous majority PF studies [10,11,12]. The EUROMACS study applied the Kormos et al. score to their dataset and reported that the Kormos et al. score had a lower confidence interval than both the CRITT and EUROMACS scores [42]. Due to the lack of echocardiographic parameters and relatively low AUC, this model was not preferred over the CRITT and Michigan scores in future comparisons. However, the study did serve an incremental role in the shift toward majority CF studies.

### 4.3. Majority CF Patient Population Models

Now that retrospective clinical data has caught up to the current trends in LVAD surgery, predictive models have been created with exclusively CF LVAD datasets. The ALMA, MCSRN, and EUROMACS scores are the most recent models developed from these datasets, possessing varying AUCs and definitions for RHF [40,41,42]. The most recent predictive score model, the MCSRN score, achieved the highest AUC of 0.89. This model’s developers, Tchantchaleishvili et al., as well as the developers of the ALMA score, Loforte et al., defined RHF solely as a patient who had a right ventricular assist device (RVAD) implanted postoperatively [40,41,42]. This differs from the EUROMACS definition, which defined RHF as a weighted hierarchy of patients that had an RVAD implanted, continuous inotropic support for ≥14 days, or NO ventilation for ≥ 48 h [42]. While all risk scores focused on early RHF, Tchantchaleishvili et al. specifically focused on acute RHF, meaning patients who required an RVAD after their hospital stay were not accounted for in the RHF group [41]. Loforte et al. and Soliman et al. accounted for all patients who developed RHF within 30 days of LVAD implantation. These degrees of exclusivity of definitions parallel the coordinate AUCs of each study. Compared to the exclusive MCSRN’s AUC of 0.89, ALMA and EUROMACS scores had AUCs of 0.77 and 0.70, respectively [40,42]. Because inotropic support and nitric oxide ventilation are typically initiated prior to RVAD implantation—owing to their less invasive nature—it is much more challenging to predict this intermediate stage of less severe RHF. In order to fairly compare these three different predictive models, a consensus must be formed on what defines RHF. Regardless, all three models performed superiorly compared to the CRITT and Michigan scores.

The parameters used by the ALMA, EUROMACS, and MSCRN scores included all modalities: laboratory, clinical, hemodynamic, and echocardiographic (Table 3). The use of echocardiographic parameters remained limited. Similar to the CRITT score, Loforte et al. designed the ALMA score with equal weights, allowing for the simple evaluation of risk once all parameters have been obtained. The use of the MELD-XI score (Equation (1)) prevents bedside calculation due to this nested equation if not previously computed [48].

(1) $$\mathrm{MELD}-\mathrm{XI}\;=5.11\times \;\ln\left(\mathrm{total}\;\mathrm{bilirubin}\right)+11.76\times \;\ln\left(\mathrm{creatinine}\right)+9.44$$

This causes all models to be similarly useful in cardiogenic shock. The MCSRN equation (Equation (2)) also uses logarithmic computation to achieve its score:

(2) $$\begin{array}{cc} \mathrm{MCSRN}\;= & 0.2944\times \;\log\left(\mathrm{HR}\right)\;-\;4.4917\times \;\log\left(\mathrm{Albumin}\right)\\ & \;+\;1.2029\times \;\log\left(\mathrm{BUN}\right)\;+\;1.0599\times \;\log\left(\mathrm{WBC}\right)\\ & \;-\;1.0364\times \;\log\left(\mathrm{CI}\right)\;+\;0.8213\times \;\mathrm{numeric}\;\mathrm{TR}\;\mathrm{severity} \end{array}$$

Interestingly, Tchantchaleishvili et al. did not include the pulmonary arterial pulsatile index (PAPi) in the multivariate analysis, even though it scored the second highest AUC of 0.78 on the dataset, higher than the CRITT score of 0.74 [41]. Incorporation of this hemodynamic parameter may increase the accuracy of the MCSRN score. 

Most previously described predictive score models (CRITT, Michigan, etc.) had patient populations numbering between 200 to 300 patients [10,11,12,44]. Other than the ALMA score, which had a patient population of 258, these new models (EUROMACS and MCSRN) had significantly larger patient populations, 2988 and 734, respectively [40,41,42]. These large patient populations reduce sampling error, which enables a higher degree of generalization if these scores are applied to other datasets. Additionally, the considerably large sample group of the EUROMACS models provided a smaller confidence interval when compared to the ALMA and MCSRN models, ± 0.03 compared to ± 0.17 and ± 0.07 [40,41,42], respectively. In future research, these models should be tested on identical patient populations with homogenous definitions for RHF in order to test these models’ generalizability and accuracy.

## 5. Modern Approaches to Post-LVAD Right Heart Failure Prediction

### 5.1. Imaging-Based Parameters

Echocardiographic methods presented an additional modality for increasing model predictive power. While current models have experimented with tricuspid regurgitation measurements, new echocardiography parameters could provide additional insight into preoperative pathophysiologies of the heart that cannot be measured with other modalities. Additionally, echocardiographic measurements are less invasive than most hemodynamic parameters incorporated into recent models.

#### 5.1.1. Longitudinal Strain

Speckle-tracking echocardiography is a newer echocardiographic technique that detects abnormal heart motion by identifying and tracking small speckles in the heart’s myocardial walls [49]. This technique has allowed for the strain of the right ventricle to be measured and incorporated into prediction models. Multiple studies have shown right ventricular global longitudinal strain (GLS) to have predictive value for post-LVAD RHF. This predictive power has been shown both through incremental addition to pre-existing models as well as independent analysis [45,46,50,51]. Impressive AUCs of 0.87, 0.86, 0.85, and 0.77 have been shown in these studies. In Liang et al.’s study, GLS was shown to have an independent AUC of 0.85 preoperatively [51]. This result has been corroborated through Kato et al.’s study, which showed GLS to have an independent AUC of 0.745 [50]. 

Despite these advances, there are several limitations of GLS that must be acknowledged before comparing it against other models and parameters. The GLS studies had patient populations below 250, with a majority below 100 patients [45,46,50,51]. In addition, all of these studies were single-center studies, unlike the EUROMACS and MCRN risk score models [41,42]. Therefore, the generalizability of these approaches remains to be evaluated. Like many other studies, the definitions of RHF were not standardized across these papers. Since longitudinal strain is a load-dependent measurement, patients who received extracorporeal membrane oxygenation during the window for echocardiography could not be included in these studies [46].

In addition to these limitations, there is disagreement over the specific type of longitudinal strain measurement that provides the greatest predictive power. GLS, free-wall longitudinal strain, and peak systolic medial free-wall longitudinal strain have all been investigated [45,46,50,51,52,53,54]. Liang et al. found that global strain had a better odds ratio than segmented strain [51]; however, Boegerhausen et al. found that segmented analysis of the free wall had more predictive power than global strain measurements [52]. Regardless, both papers agreed that free-wall strain was more useful than septal strain in segmented analysis.

Where segmented analysis has been favored, free-wall right ventricular longitudinal strain (fwLS) has been shown to have significant predictive value. AUCs of 0.93 and 0.71 have been shown with independent application of fwLS (Table 4) [52,53]. These values follow the strong negative correlation that has been observed between fwLS and RVWSI, a hemodynamic parameter used by the ALMA score [40,54]. fwLS was shown to have a higher predictive power of RHF compared to RVSWI [54]. In a study conducted on 10 patients who received the Jarvik 2000 LVAD, a CF LVAD, fwLS was shown to have the highest AUC of 0.93 compared to other clinical, echocardiographic, and hemodynamic parameters after multivariate analysis [55]. These patterns may make fwLS a suitable replacement for older PF-centric parameters.

The primary limitation of all longitudinal strain parameters is their requirement for high-quality imaging and separate analysis using specialized software. In one of the largest datasets where longitudinal strain was investigated, 136 of the original 497 patients were excluded from the study due to suboptimal imaging [45]. For longitudinal strain parameters to be widely applicable, optimized imaging must be clinically standard. And, while almost all hospitals have echocardiographic devices, experience with this analysis and software is limited and must be expanded for this measurement modality to be included in future useful predictive models.

#### 5.1.2. Fractional Area Change (FAC)

Right ventricular fractional area change (FAC) is a global systolic measurement calculated from the difference in end-diastolic area and end-systolic area divided by the end-diastolic area. While FAC remains to be used as a method to evaluate right ventricular function, findings on its predictive power for post-LVAD RHF have been incongruous. Cacioli et al. used FAC in a multivariable model, which achieved an AUC of 0.949; however, they did not provide an independent AUC like they did for their other parameter, PAPi [56]. While addressing limitations, Calioli et al. did recognize that the lack of speckle-tracking echocardiography may have impacted their findings. Future papers that did have access to speckle tracking data found that strain measurements were more prognostically useful than FAC [51,57]. In Belavia et al.’s meta-analysis, FAC had a low effect size in discriminating between non-RHF and RHF groups across eleven studies with an I^2^ value of 18.1. Several other papers have found that FAC was not significantly associated with RHF or had a poor specificity of 52% [50,51,52,53]. While FAC may remain useful for clinicians who do not have access to speckle-tracking echocardiography software, its predictive ability still remains to be shown.

#### 5.1.3. Cardiac MRI and 3-D Echocardiography

Since many advanced-stage heart failure patients have previously implanted hardware (pacemaker, implanted cardioverter defibrillator, etc.), three-dimensional echocardiography presents another modality to supplement cardiac MRIs. Multiple meta-analysis studies have been conducted to validate 3-D echocardiography against cardiac MRIs [58,59]. Right ventricular ejection fraction (RVEF), commonly measured through this modality, could be measured through 3-D echocardiography instead. The prognostic value of RVEF has been shown through Nagata et al.’s study, connecting this value to poor cardiovascular outcomes and cardiovascular-related death [60]. Regardless, additional advances in the average cardiovascular physician’s experience with 3-D echocardiographic analysis must increase before this parameter can be incorporated.

### 5.2. Hemodynamic Parameters

#### Pulmonary Artery Pulsatile Index (PAPi)

The pulmonary artery pulsatile index is a ratio measurement between the pulmonary artery pulse pressure and the right atrial pressure. This value is calculated from systolic pulmonary artery pressure–diastolic pulmonary artery pressure/right atrial pressure obtained during right heart catheterization. Several papers have used PAPi independently or in multivariable models to predict post-LVAD RHF. AUCs of 0.949, 0.87, 0.85, and 0.77 have been associated with PAPi-incorporated models [45,53,56,61]. In Stricagnoli et al.’s study, various hemodynamic parameters were evaluated, and PAPi was found to be the most statistically significant hemodynamic parameter correlated with post-LVAD RHF [53]. As an independent predictor of RHF, PAPi was found to have an AUC of 0.85 with their dataset. When compared to the RA/PCWP ratio, another statistically significant hemodynamic parameter, PAPi was found to have higher predictive power; they proposed that the difference in predictive power was likely due to PAPi’s smaller dependence on left heart influence [53]. This finding built upon Kang et al.’s finding that RA/PCWP’s relationship to RHF was significantly reduced when patients were prescribed inotropes at the time of catheterization [61]; conversely, PAPi was found to have higher predictive power when patients were given inotropes at the time of catheterization when compared with non-inotrope patients.

This relationship drove Cacioli et al. to investigate how a vasodilator challenge would affect the predictive power of PAPi. The vasodilator challenge is a diagnostic tool used to evaluate the reversibility of pulmonary arterial hypertension. In Cacioli et al.’s study, the vasodilator challenge was accomplished through the slow up-titration of sodium nitroprusside (NTP) to a systolic blood pressure of 85 mmHg or the maximum tolerated effect. From their study, they found that post-NTP PAPi was an independent predictor of post-LVAD RHF whereas baseline PAPi was not. Additionally, when post-NTP PAPi was paired with the EUROMAC predictive score model, there was a statistically significant increase in predictive accuracy [56].

While PAPi, and specifically postvasodilator PAPi, seems promising, these studies were single center and retrospective. Their patient populations were below 250 and had varying definitions for post-LVAD RHF [45,53,56,61]. Lim and Gustafsson have also seen wide variations in PAPi values based on patient populations’ pathophysiologies [62]. Isaza et al. have proposed that these variations may be bifurcated into two unique groups: patients with impaired RV systolic function and low RA pressure or patients with preserved RV systolic function and high PA pulse pressure. They have proposed and found utility using global longitudinal strain to distinguish between these two groups with an AUC of 0.949 [45]. The combination of these parameters is applied similarly to Wang et al.’s decision tree [44], allowing for similarly easy clinical application.

Nevertheless, postvasodilator PAPi may provide additional benefits to pre-existing predictive score models, especially in conjunction with longitudinal strain data. The ALMA score has already incorporated baseline PAPi into its model [40], and the EUROMACS score could substitute the less accurate RA/PCWP for postvasodilator PAPI [42].

### 5.3. Bayesian and Machine Learning (ML) Models

Artificial intelligence has revolutionized data analysis and prediction across almost all fields, especially biostatistics. Multiple researchers have used these techniques in retroactive studies to predict RHF. Different approaches have been applied to post-LVAD RHF prediction in CF patient populations, separated based on their input and their ML architecture. Shad et al. used two parallel spatiotemporal streams of data from echocardiography videos to train their ML model [63]. Their AI system outperformed both the quantitative echocardiographic parameters and clinical predictions for RHF. These echocardiographic parameters included left ventricular end-systolic area (LV-ESA), right ventricular end-diastolic area RV-EDA, RV ejection fraction, and RV end-systolic area (RV-ESA), TAPSE, and RV strain. Interestingly, RV longitudinal strain was the most predictive of the quantitative echocardiographic parameters. For the ML system, they applied fourteen different ML architectures, with the Resnet-152 + optical flow achieving the highest AUC of 0.749. This study was the most comprehensive application of different ML architectures to post-LVAD RHF prediction [63].

The STOP-RVF score, developed by Taleb et al., applied supervised ML to achieve an AUC of 0.729. Unlike Shad et al., the STOP-RVF score was generated from zero-dimensional quantitative clinical, laboratory, hemodynamic, and echocardiographic parameters [63,64]. During data preprocessing, eleven variables were selected, including RA/PCWP. Although PAPi was associated with post-LVAD RHF, RA/PCWP was found to have a higher C-index for their dataset. Additionally, speckle-tracking echocardiography parameters like RV longitudinal strain were absent from their study [64]. The inclusion of post-vasodilator PAPi and longitudinal strain may increase the accuracy of this model in future experimentation, as these parameters have shown high independent predictive power.

Loghmanpour et al. favored a tree-augmented naive Bayesian architecture. With their model, they were able to derive an impressive degree of stratification, predicting acute, early, and late post-LVAD RHF [65]. For all three models, reported specificity surpassed 98%; however, sensitivity was significantly lower. They reported AUCs of 0.903, 0.835, and 0.883 for acute (<48 h), early (48 h to 14 days), and late (>14 days) RHF, respectively. These AUCs were achieved on a dataset of over 10,000 patients, significantly dwarfing all other patient populations reviewed in this paper [65]. On the same dataset, the Michigan score and the Drakos et al. score achieve AUCs of 0.498 and 0.547, respectively. Since these older scores (Michigan and Drakos) were created with a majority of PF patient populations, it is expected that they would not perform well on the patient population Loghmanpour et al. trained their model on, which had 9976 patients with CF LVADs and only 933 with PF LVADs [10,11,65]. Unlike the Shad et al. and Taleb et al. studies, no echocardiographic parameters were included in this study [63,64]. Due to the large patient population of the Loghmanpour et al. study, a ten-fold validation was applied [65]. The inclusion of postvasodilator PAPi and longitudinal strain parameters may incrementally increase the accuracy of their models, as shown with the EUROMACs and CRITT score in Cacoli et al.’s study [56]. Of the ML approaches, Loghmanpour et al.’s is the most auspicious, with the high predictive power and ability to differentiate between RHF prognosis. 

Machine learning approaches to predicting RHF have several general benefits and drawbacks that must be addressed. Of the three previously discussed studies, all included patient populations exceeding 700 patients in their analysis (Table 5). Despite the comprehensiveness of their population size, ML studies are constrained by their ability to translate to the clinic. Notably, of the studies discussed, only the study by Shad et al. provided open-source code [63], which is crucial for enabling other institutions to develop, validate, and replicate similar clinical tools. Compared to the other predictive approaches reviewed, ML models present greater challenges in clinical application and inter-institutional collaboration.

### 5.4. Mechanistic Modeling

Despite healthcare’s shift toward increasingly tailored and patient-specific approaches, post-LVAD RHF predictive modeling has largely remained using standardized models based on population trends. While these models do have merit, their framework is applied without respect to the unique cardiovascular physiology of each patient. Mechanistic modeling may be a method to allow for this tailored-medicine perspective to reach LVAD patients. Mechanistic modeling uses preoperative patient-specific data to construct an in silico and in vitro patient-specific model; with this tailored model, “virtual treatments” can then be applied where clinicians are able to evaluate the consequences of treatments without risk to patients [64,66,67,68,69,70,71,72,73,74]. In the field of post-LVAD RHF prediction, lumped parameter models are tuned with preoperative hemodynamic parameters (cardiac output, systolic and diastolic blood pressures, vascular resistances, and ventricular contractilities) to create a digital twin of the patient. Once the model is tuned to the patient’s preoperative state, the addition of an LVAD can be modeled to investigate how the patient’s cardiovascular health is impacted.

Kung et al.’s research group recently proposed combining physical fluid experimentation with lumped parameter network simulation to provide a mechanistic framework for predictive modeling; this approach, named the Physiology Simulation Coupled Experiment (PSCOPE) [75,76,77], allows for the predictive model to account for patient-specific cardiovascular nuances. Using the latest INTERMACS guidelines for post-LVAD RHF, such a model was able to accurately predict the absence of RHF in the three patient cases included in a clinical model validation study [78]. However, due to the limited sample size, its applicability to a broader patient population remains uncertain. Nonetheless, this novel approach contrasts with the correlative nature of current methods by offering a mechanistic foundation while also yielding simulated metrics that can serve as valuable features in machine learning approaches. However, further evaluation in a larger patient population is warranted.

## 6. Conclusions

As the devices, preoperative measurement modalities, and patient populations of LVAD implantation continue to evolve over time, predictive risk score models have progressed and incorporated these advances as they become available. Current predictive score models like the EUROMACS, MCSRN, and ALMA models have unique properties and various definitions of RHF, which contribute to their varying specificities and sensitivities [40,41,42]. Homogeneity of RHF definitions should be pursued to allow for better generalizability validation. Additionally, the Michigan score should no longer be compared against current models as that score’s patient population is vastly different from current populations and, therefore, can no longer provide valuable commentary on a current model’s validity. Investigation into whether the global or free-wall longitudinal strain is a better metric for prediction should be conducted. In addition, post vasodilator PAPi and longitudinal strain should be applied to larger multicenter patient populations to determine the replicability of the smaller studies reviewed in this paper. The application of machine learning and mechanistic modeling holds exciting potential for continued exploration. The development of tools for the quick assessment of RHF risk should be emphasized to ensure clinicians can effectively apply these models in practice.

## Figures and Tables

**Table 1:** Clinical findings related to RHF [36].

Type of Finding	Cutoff
Ascites	Yes/No
Functionally limiting peripheral edema	>2+
Elevated estimated jugular venous pressure at least halfway up the neck in an upright patient	Yes/No
Elevated measured central venous pressure or right atrial pressure	≥16 mm Hg

**Table 2:** Clinical manifestations associated with RHF [36].

Type of Finding	Cutoff
Renal failure with serum creatinine	>2 × baseline values
Liver injury with an elevation	2 × upper limit normal in aspartate aminotransferase/alanine aminotransferase, or total bilirubin > 2.0
SVO_2_	<50%.
Cardiac index	<2.2 L/min/m^2^
Reduction in pump flow	>30% from the previous baseline in the absence of mechanical causes

**Table 3:** Majority CF patient population model parameters [40,41,42].

Parameter Modality	Parameter	Cutoff	Risk Score
Clinical	LVAD as DT	Yes/No	ALMA
	MELD-XI	>17	ALMA
	MII	Yes/No	EUROMACS
	INTERMACS	≥3	EUROMACS
Hemodynamic	PAPi	<2	ALMA
	RVSWI	<300 mmHg/mL/m^2^	ALMA
	HR	CoE2	MCSRN
	CI	CoE2	MCSRN
	RA/PCWP	≥0.54	EUROMACS
Laboratory	Albumin	CoE2	MCSRN
	Creatinine WBC Hb	CoE2 CoE2 ≤10 g/dL	MCSRN MCSRN EUROMACS
Echcardiographic	RV/LV ratio	>0.75	ALMA
	TR Severity	CoE2	MCSRN
	RVD	Yes/No	EUROMACS

CoE2 Component of Equation (2), WBC white blood count, Hb hemoglobin, MELD-XI score model for end-stage liver disease excluding international normalized ratio, RVD right ventricle dysfunction, MII multiple intravenous inotropes, INTERMACS interagency registry for mechanically assisted circulatory support class, PAPi pulmonary artery pulsatility index, RVSWI right ventricular stroke work index, HR heart rate, CI cardiac index, RA/PCWP right atrial/pulmonary capillary wedge pressure ratio, RV/LV ratio right to left ventricular end-diastolic diameter ratio, TR severity tricuspid regurgitation severity.

**Table 4:** Imaging-based studies.

Study	Patient Population	Parameter	AUC
Isaza et al. [45]	246	PAPi, GLS, Michigan score	0.87
Liang et al. [51]	55	GLS	0.85
Cacioli et al. [56]	54	FAC, post-NTP PAPi, PASP	0.949
		post-NTP PAPi	0.75
Kato et al. [50]	24	GLS, S′, RV E/E′	0.8475
		GLS	0.745
Grant et al. [46]	117	GLS, Michigan score	0.77
Boegerhausen et al. [52]	46	PS-fwLS, Hb, PCT, RVSP, Pre-CVP	0.92
		PS-fwLS	0.71
Kang et al. [55]	85	PAPi	0.77
Stricagnoli et al. [53]	38	PAPi	0.85
		fwLS	0.93

PAPi pulmonary artery pulsatility index, GLS global longitudinal strain, FAC fractional area change, post-NTP postsodium nitroprusside administration, PASP pulmonary artery systolic pressure, S′ peak systolic velocity of the RV free wall at the tricuspid annulus, E peak early trans-tricuspid filling velocity, E′ early diastolic velocity of the RV free wall at the tricuspid annulus, PS-fwLS peak systolic free-wall longitudinal strain, Hb hemoglobin, PCT procalcitonin, RVSP right ventricular systolic pressure, Pre-CVP central venous pressure intraoperative before insertion of the heart–lung machine’s cannula.

**Table 5:** Bayesian and machine learning (ML) models summary.

Study	Type of ML Architecture	Patient Population	AUC
STOP-RVF score [64]	Supervised machine learning using multiple imputations of chain equations imputed model coefficients	Derivation = 798	AUC = 0.729
		Validation = 327	
Shad et al. [63]	Two-stream fusion 152-layer 3D residual network with bottlenecks incorporated within the residual block	Training = 467	AUC = 0.749
		Testing = 121	
		Validation = 135	
Loghmanpour et al. [65]	Tree-augmented naïve Bayesian architecture	Training = 9818Testing = 1091	Acute RHF = 0.903
			Early RHF = 0.835
			Late RHF = 0.883

## Supplementary Materials

The following supporting information can be downloaded at: https://www.mdpi.com/article/10.3390/jcdd12070240/s1, Table S1: Definitions of post-LVAD right heart failure; Table S2: Majority PF patient population model parameters [10,12].

## Abbreviations

The following abbreviations are used in this manuscript:

RHF	Right heart failure
LVAD	Left ventricular assist device
PF	Pulsatile-flow
CF	Continuous-flow
LV	Left ventricle
RV	Right ventricle
BNP	Brain natriuretic peptide
ECM	Extracellular matrix
RVSWI	Right ventricular stroke work index
AUC	Area under the curve
RVAD	Right ventricular assist device
HR	Heart rate
BUN	Blood Urea Nitrogen
WBC	White blood count
CI	Cardiac index
TR	Tricuspid regurgitation
GLS	Global longitudinal strain
fwLS	Free-wall longitudinal strain
FAC	Fractional area change
PAPi	Pulmonary artery pulsatile index
Post-NTP	Post-sodium nitroprusside administration
iNO	Inhaled nitric oxide
PASP	Pulmonary artery systolic pressure
S’	Peak systolic velocity of the RV free wall at the tricuspid annulus
E	Peak early trans-tricuspid filling velocity
E’	Early diastolic velocity of the RV free wall at the tricuspid annulus
PS-fwLS	Peak systolic free-wall longitudinal strain
Hb	Hemoglobin
PCT	Procalcitonin
RVSP	Right ventricular systolic pressure
Pre-CVP	Central venous pressure intraoperative before insertion of the heart–lung machine’s cannula
PCWP	Pulmonary capillary wedge pressure
MRI	Magnetic resonance imaging
3-D	Three dimensional
RVEF	Right ventricular ejection fraction
RA/PCWP	Right atrial pressure to pulmonary capillary wedge pressure ratio
ML	Machine learning
LV-ESA	Left ventricular end-systolic area
RV-ESA	Right ventricular end-diastolic area
TAPSE	Tricuspid annular plane systolic excursion
MCS	Mechanical circulatory support
PSCOPE	Physiology simulation coupled experiment
