# Supplementary material for: Prediction of Right Heart Failure in LVAD Candidates: Current Approaches and Future Directions
Source: J Cardiovasc Dev Dis. 2025 Jun 23;12(7):240. doi: 10.3390/jcdd12070240 (PMC12295032; doi:10.3390/jcdd12070240)
Supplement: Supplementary file 1 [file jcdd-12-00240-s001.zip › jcdd-3578603-supplementary.pdf]

Table S1. Definitions of post-LVAD right heart failure.

Scoring System	Definitions of post-LVAD RHF
Matthews et al. score (Michigan score) [11]	Need for postoperative intravenous inotrope support for >14 days, inhaled nitric oxide for ≥48 h, right-sided circulatory support, or hospital discharge on an inotrope.
Drakos et al. score (Utah score) [10]	Need for inhaled nitric oxide for ≥48 h or intravenous inotropes for >14 days and/or right ventricular assist device implantation.
Fitzpatrick et al. score (Pennsylvania score) [12]	Required RVAD support
Pittsburgh decision tree score [39]	Implantation of RVAD
CRITT score [13]	Elevated central venous pressure (CVP; >18 mm Hg) with depressed cardiac index (<2 L/min/m^2) in the absence of elevated pulmonary capillary wedge pressure (PCWP; >18 mm Hg), requiring RVAD implantation or requirement of prolonged (>1 week) nitric oxide or inotropic therapy.
Kromos et al. score [14]	RVF was defined in the HeartMate II clinical trial as either the need for an RVAD in addition to the LVAD (group 1), continuous inotropic support for at least 14 days after implantation (group 2), or late inotropic support starting 14 days after implantation (group 3). Data from groups 1 and 2 were combined to form an early RVF group, whereas group 3 patients were examined separately (late RVF group).
EUROMACS score [37]	Severe postoperative RHF, defined as receiving short- or long-term right-sided circulatory support, continuous inotropic support for ≥14 days, or NO ventilation for ≥48 h
ALMA score [35]	The primary outcome was severe RVF within 30 days of LVAD implantation, defined as receiving short- or long-term right-sided circulatory support despite maximal dosage of continuous inotropic support and NO ventilation. The secondary outcome was all-cause mortality.
MCSRN score [36]	We chose to use an RVAD as the primary outcome due to varying definitions of RVF at our institutions. These patients had acute severe RVF.
Isaza et al. [40]	Inotrope support) for >14 days, or unplanned RV assist device insertion after LVAD implantation
Liang et al. [65]	Acute RHF was defined in accordance with the newly updated MCS–ARC report as the need for implantation of a temporary or durable RVAD (including ECMO) concomitant with LVAD implantation (RVAD implanted prior to the patient leaving the operating room).<br>Early post-implant RHF was defined as the need for implantation of a temporary or durable RVAD (including ECMO) within 30 days following LVAD implantation for any duration of time or failure to wean from inotropic or vasopressor support or inhaled nitric oxide within fourteen days following LVAD implantation or having to initiate this support within thirty days of implant for a duration of at least 14 days.
Cacioli et al. [51]	The need for short- or long-term, right-sided circulatory support, continuous inotropic support for ≥14 days, or inhaled nitric oxide (iNO) ventilation for ≥48 h postoperatively.

Kato et al. [46]	The need for RVAD, inotropic support at 14 days after surgery, or inhaled or oral pulmonary vasodilators (iloprost, inhaled nitric oxide, or sildenafil) at 14 days after surgery.
Grant et al. [41]	Unplanned insertion of an RVAD or the use of an intravenous inotrope for >14 days postoperatively
Boegerhausen et al. [47]	The need for an RV assist device or the requirement of inhaled nitric oxide or inotropic therapy for >1 week any time after LVAD implantation in the presence of symptoms and signs of persistent RV dysfunction, such as central venous pressure (CVP) >18 mmHg with a cardiac index <2.3 L/min/m^2 in the absence of elevated left atrial or pulmonary capillary wedge pressure >18 mmHg, cardiac tamponade, ventricular arrhythmias or pneumothorax
Kang et al. [52]	Elevated central venous pressure (CVP; >18 mm Hg) with depressed cardiac index (<2 L/min/m^2) in the absence of elevated pulmonary capillary wedge pressure (PCWP; >18 mm Hg), requiring RVAD implantation, or requirement of prolonged (>1 week) nitric oxide or inotropic therapy.
Stricagnoli et al. [48]	Elevated CVP with depressed cardiac index (<2 L/min/m^2) in the absence of elevated PCWP (<18 mmHg), requirement for right VAD implantation, or requirement for prolonged (4 days–1 week) inhaled nitric oxide or inotropic therapy.
STOP-RVF score [58]	Need for inotrope therapy for greater than 14 days and/or right-sided circulatory support (surgically or percutaneous-implanted MCS) within 30 days postoperatively.
Shad et al. [59]	Prolonged pos-implant inotropes, inhaled nitric oxide, or intravenous vasodilators continued beyond postop day 14 following LVAD implant OR need for right ventricular assist device at any time following LVAD implant OR death during the LVAD implant's hospitalization with RHF as the primary cause.
Loghmanpour et al. [42]	Central venous pressure >18 mmHg with a cardiac index <2.0 L/min/m^2, right ventricular assist device implantation OR use of inhaled nitric oxide or inotropic therapy for a duration of more than 1 week at any time after LVAD implantation, Absence of elevated left atrial or pulmonary capillary wedge pressure (>18 mmHg), tamponade, ventricular arrhythmias or pneumothorax

Table S2. Majority PF patient population model parameters [10,12].

Parameter Modality	Parameter	Cutoff	Risk Score
Clinical	LVAD as DT	Yes/No	Utah
	IABP	Yes/No	Utah
	Inotropes	Yes/No	Utah
	Obesity	Yes/No	Utah
	β blockers	Yes/No	Utah
	RVD	Yes/No	Pennsylvania
	Precardiac Surgery	Yes/No	Pennsylvania
Hemodynamic	PVR	≤1.7 wood units	Utah
	RVSWI	≤250 mmHg mL/m^2	Pennsylvania

	SBP	≤96 mmHg	Pennsylvania
Laboratory	ACEi/ARB	Yes/No	Utah
	Creatinine	≥1.9 mg/dL	Pennsylvania

ACEi/ARB angiotensin-converting enzyme inhibitors/angiotensin receptor blockers, IABP intra-aortic balloon pump, RVD right ventricle dysfunction, PVR pulmonary vascular resistance, RVSWI right ventricular stroke work index, SBP systolic blood pressure.
